# Structural health determinants and comorbidity risk factors for COVID-19 outcomes among participants in the UK Biobank.

**DOI:** 10.23889/ijpds.v7i3.1836

**Published:** 2022-08-25

**Authors:** Deborah Allen, Alejandra Vergara-Lope, Daniel Wilson

**Affiliations:** 1 University of Oxford; 2 Big Data Institute, University of Oxford

## Objectives

COVID-19 disproportionately affects older patients with pre-existing health conditions. However at the time of writing, June 2021, the relationship between COVID-19, ethnicity and deprivation is unclear. We estimated associations between individual comorbidities and local deprivation with a range of COVID-19 outcomes, in a large national cohort: UK Biobank.

## Approach

Data linkage between COVID-19 records in England with baseline participant information and secondary care diagnoses from the UK Biobank allowed estimation of associations between deprivation, comorbidities and COVID-19 outcomes.

Multivariable logistic regression models were constructed for COVID-19 test results, hospitalisations and deaths recorded between 1st January 2020 to 31st March 2021. High-risk clusters for COVID-19 were mapped using principal components analysis and minimum variance stratification, according to age, comorbidities and deprivation.

27,306 participants tested for SARS-CoV-2 were eligible for inclusion. Of these, 5,196 tested positive, 2,724 were hospitalised and 475 died.

## Results

Positive linear trends were observed between higher local deprivation and higher odds of SARS-CoV-2 infection, COVID-19 hospitalisations and deaths (p<0.001).

The odds of testing positive was 19% lower for participants with cancer (adjusted odds ratio, aOR=0.81, 0.74-0.88). Higher odds of testing positive were associated with younger age, male sex, cerebrovascular disease and more severe deprivation.

Participants with diabetes were at 42% higher odds of hospitalisation (aOR=1.42, 1.30-1.56). In addition to older age and male sex, strongest comorbidity risk factors for COVID-19 death were cerebrovascular disease, diabetes and mild liver disease with aOR of 1.78 (1.34-2.36), 1.71 (1.41-2.08) and 1.85 (1.31-2.61) respectively, after full adjustment.

Participants recruited from North-West England were in highest clusters of COVID-19 risk, whilst participants from South England were at lowest risk.

## Conclusion

The burden of COVID-19 increased at higher levels of deprivation, and the presence of specific comorbidities. Individuals in more deprived areas were at significantly higher odds of infection, hospitalisation and death from COVID-19. Several cardiovascular comorbidities and associated risk factors were associated with COVID-19 hospitalisation and death.

